# Functional linear modeling of activity data shows analgesic-mediated improved sleep in dogs with spontaneous osteoarthritis pain

**DOI:** 10.1038/s41598-019-50623-0

**Published:** 2019-10-02

**Authors:** M. E. Gruen, D. R. Samson, B. D. X. Lascelles

**Affiliations:** 1 Department of Clinical Sciences, North Carolina State University College of Veterinary Medicine, Raleigh, NC USA; 2 Comparative Pain Research and Education Center, North Carolina State University, Raleigh, NC USA; 3 Department of Anthropology, University of Toronto Mississauga, Mississauga, ON Canada; 4 Translational Research in Pain Program, North Carolina State University, College of Veterinary Medicine, Raleigh, NC USA; 5 Thurston Arthritis Center, UNC School of Medicine, Chapel Hill, NC USA; 6 Center for Translational Pain Research, Department of Anesthesiology, Duke University, Durham, NC USA

**Keywords:** Musculoskeletal system, Osteoarthritis

## Abstract

In humans, pain due to osteoarthritis has been demonstrated to be associated with insomnia and sleep disturbances that affect perception of pain, productivity, and quality of life. Dogs, which develop spontaneous osteoarthritis and represent an increasingly used model for human osteoarthritis, would be expected to show similar sleep disturbances. Further, these sleep disturbances should be mitigated by analgesic therapy. Previous efforts to quantify sleep in osteoarthritic dogs using accelerometry have not demonstrated a beneficial effect of analgesic therapy; this is despite owner-reported improvements in dogs’ sleep quality. However, analytic techniques for time-series accelerometry data have advanced with the development of functional linear modeling. Our aim was to apply functional linear modeling to accelerometry data from osteoarthritic dogs participating in a cross-over non-steroidal anti-inflammatory (meloxicam) drug trial. Significant differences in activity patterns were seen dogs receiving drug (meloxicam) vs. placebo, suggestive of improved nighttime resting (sleep) and increased daytime activity. These results align with owner-reported outcome assessments of sleep quality and further support dogs as an important translational model with benefits for both veterinary and human health.

## Introduction

In humans, clear evidence exists that chronic pain interferes with sleep^1^. Sleep disturbances decrease quality of life, are associated with higher anxiety and depression^2^, and worsen chronic pain symptoms^3^. A common cause of chronic pain is osteoarthritis (OA). Several studies have reported insomnia^4,5^, decreased sleep quality^6^, and increased self-reporting of pain^7^ in people with OA. Dogs also suffer from OA that is pathologically and symptomatically similar to humans. These similarities have led to the dog’s emergence as a good naturally-occurring model for understanding human arthritis pain^8,9^. An improved understanding of the association between OA and sleep in dogs will enhance their use as a model for human OA.

In humans, sleep quality is often measured objectively using actigraphy^7^; lower activity counts indicative of less movement are presumed to reflect higher quality sleep. Disturbances of sleep occur due to pain states, but interestingly there are little data on the use of actigraphy to monitor sleep quality in relation to pain relief. Analgesia-associated modification of sleep in dogs with OA has been previously evaluated by our laboratory using accelerometry and an owner-completed sleep quality questionnaire, the Sleep and Night Time Restlessness Evaluation (SNoRE)^10^. The SNoRE is a six-item instrument which asks owners to rate comfort and quality features of their dog’s sleep. In this study, dogs with osteoarthritis wore accelerometers over a five-week period; they received meloxicam (Metacam®, Boehringer-Ingelheim) and placebo, each for two weeks, in a randomized crossover design. Using the SNoRE questionnaire, the study found that dogs receiving meloxicam had improved sleep quality when compared to placebo; however, no difference in mean accelerometry counts was observed. Questionnaire items that demonstrated the highest responsiveness to meloxicam treatment regarded twitching, dreaming, shifting position, and vocalizing. We concluded that while the SNoRE questionnaire demonstrated responsiveness validity, criterion validity of the instrument could not be inferred due to the lack of a change in activity as measured with accelerometry [10]. This previous study has an important limitation; however, accelerometry data were collected every minute and then averaged across multiple hours in the statistical analysis. This resulted in an inability to detect short-term changes in behavior reported by owners (e.g., shifting position).

Functional data analysis, particularly functional linear modeling (FLM), has been developed specifically to evaluate actigraphy time-series data. This analytical approach has led to important discoveries about chronotype variation and sleep-wake regulation across human groups living in natural, non-industrial environments^11–14^. In animals, functional data analysis has been used to demonstrate subtle activity changes in cats with degenerative joint disease^15^. This latter study in cats highlights the potential applications for functional data analysis in studies of pain and activity, but to date, no studies in dogs or cats have used FLM to assess the impact of pain relief on activity or sleep in a chronic pain state.

Pain-related sleep disturbance in humans is considered to be due to spontaneous pain as opposed to movement or activity-related pain; self-reported spontaneous pain in humans has been very difficult to measure in animal models. Assessment of sleep quality in the canine osteoarthritis model may provide a highly relevant method to assess the effectiveness of analgesics on spontaneous pain. In the present study, we use FLM to reanalyze our data from the SNoRE study to evaluate the utility of using FLM to measure improved sleep quality due to the alleviation of spontaneous pain, and to assess the validity of our questionnaire.

## Results

### Subjects

Fifteen dogs (10 spayed females and 5 neutered males) had complete activity data sets and were included in the analysis. Dogs had a mean (±SD) age of 10.29 ± 2.48 years, weight of 31.53 ± 5.39 kgs, Canine Brief Pain Inventory (CBPI) pain score of 3.85 ± 1.5.

### SNoRE questionnaire data

As previously reported^10^, the overall score on the SNoRE instrument detected a positive improvement due to the NSAID (p = 0.001) and detected a difference between the NSAID and placebo (p = 0.041). Table 1 details the change from baseline score for each questionnaire item and for the overall score.

**Table 1:** Results of the SNoRE questionnaire for each individual question as well as total score for all six questions (6Q) and for a modification using only five questions (5Q) with the removal of Question 6. Results are shown for each treatment period compared to baseline, and comparing treatment with an NSAID (meloxicam) to placebo.

	NSAID – Baseline		Placebo – Baseline		NSAID - Placebo	
	Mean difference	*p*-value	Mean difference	*p*-value	Mean difference	*p*-value
Q1:Movement	−1.00	0.038	−0.33	0.554	−0.67	0.313
Q2:Twitching	−1.20	0.028	−0.20	0.638	−1.40	**0.012**
Q3: Dreaming	−1.80	**<0.001**	−0.40	0.32	−1.40	**<0.001**
Q4:Shifting position	−1.13	0.059	−0.13	0.860	−1.00	0.165
Q5: Vocalizing	−1.07	**0.010**	−0.60	0.082	−0.47	0.131
Q6: Pacing	0.27	0.452	0.33	0.371	−0.07	0.915
Total (Q 1–5)	−6.20	**0.001**	−1.27	0.456	−4.93	**0.029**
Total (6Q)	−6.47	**0.001**	−0.93	0.616	−5.53	**0.041**

### Linear mixed-effects model

Our results replicated the previously reported finding: using this method we found no effect of treatment on nighttime activity. Weekend and weekday were included as covariates as previous work has shown a difference in activity on weekdays versus weekends in dogs^29^. This model found that only weekend versus weekday was significantly associated with nighttime activity; regardless of treatment, dogs were more active on weekend nights than weekdays. Table 2 shows the results of the model for nighttime activity.

**Table 2:** Model-averaged coefficients from linear mixed-effects model evaluating covariate effects on canine nighttime activity.

	Estimate	Standard Error	z-value	P(>|z|)
Age	0.228	0.154	1.480	0.139
Weekend/Weekday	0.108	0.039	2.732	**0.006**
CBPI Score	−0.196	0.160	1.220	0.223
Sex	−0.169	0.165	1.021	0.307
Weight	−0.155	0.180	0.861	0.389
Treatment	−0.022	0.040	0.554	0.580

Significant effects were found only for day of the week (weekend activity was higher than weekday). CBPI = Canine Brief Pain Inventory.

Results for the linear mixed-effects model for daytime activity are shown in Table 3. As with the nighttime activity, weekend vs. weekday was significantly associated with daytime activity; dogs were significantly more active on weekend days than weekdays, regardless of treatment. In addition, dogs with higher baseline CBPI scores were more active during the day (p = 0.041) and male dogs were more active than female dogs (p = 0.032). The correlations of daytime activity with these two variables were modest (CBPI r = 0.21; Sex r = 0.35).

**Table 3:** Model-averaged coefficients from linear effects model evaluating covariate effects on canine daytime activity.

	Estimate	Standard Error	z-value	P(>|z|)
Age	−0.082	0.194	0.420	0.675
Weekend/Weekday	0.101	0.028	3.521	**<0.001**
CBPI Score	0.388	0.189	2.040	**0.041**
Sex	0.414	0.192	2.148	**0.032**
Weight	0.218	0.212	1.026	0.305
Treatment	0.016	0.028	0.550	0.582

Significant effects were found for day of the week (weekend activity was higher than weekday), CBPI score (dogs with higher baseline CBPI scores were more active), and sex (males were more active than females).

### Functional linear modeling

**Figure 1:**
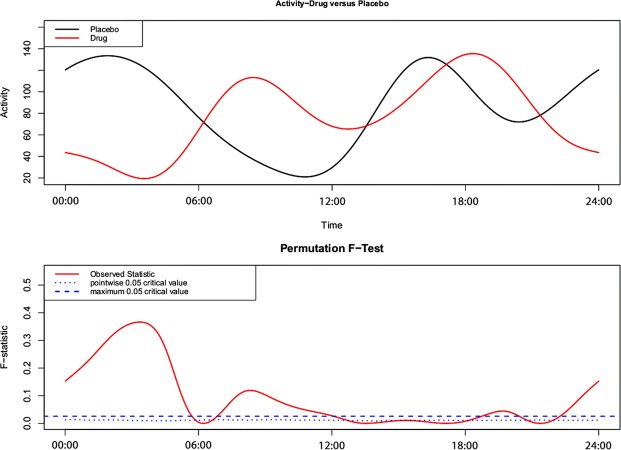
A functional linear modeling comparison between the 24-hour sleep-wake pattern of placebo and drug trials. The bottom panel illustrates the point-wise critical value (dotted line). This is the proportion of all permutation *F* values at each time point at the significance level of 0.05. When the observed *F*-statistic (solid line) is above the dotted line, it is concluded the two groups have significantly different mean circadian activity patterns at those time points.

In contrast to the linear mixed-effects model, the functional linear model found significant differences in activity when the dogs were receiving meloxicam versus placebo. Figure 1 shows the group level circadian activity pattern, with red representing the drug group and black representing the placebo group. This plot shows a clear separation of the mean circadian activity and identifies the time periods when the curves differ between groups: (23:00–06:00, 07:00–12:00, 21:00–22:00). In general, differences in circadian activity were apparent, with the drug group characterized by decreased nighttime activity between 23:00–06:00, and increased daytime activity between the periods of both 07:00–12:00 and 21:00–22:00. In other words, we found that when receiving meloxicam, relative to placebo, the dogs had greater nighttime resting and more pronounced activity during the day.

## Discussion

In this study, when we used functional linear modeling, we found a robust and significant difference (previously masked when applying a more traditional technique) in the pattern of nighttime and daytime activity in dogs receiving meloxicam, compared to placebo. The traditional technique of analyzing high-frequency longitudinal activity data uses summary values, averaging activity counts over large periods of time; this sacrifices the detail available in the data. Importantly, summary statistical approaches do not allow for understanding patterns of activity. Functional linear modeling (FLM) is designed specifically to address these limitations; the granularity of the data is harnessed to allow analysis of the pattern of activity across the day^16^. Using this approach, we found improved sleep, as defined by decreased nighttime activity, and this was supported by the owners’ assessment of quality of sleep of their pet. To our knowledge, FLM has not been applied to activity data from any species in relation to pain and pain relief previously.

We first re-evaluated the traditional technique by replicating the previous study’s findings using linear mixed-effects modeling^10^. Of the tested covariates, only activity on the weekend, relative to weekday, was significantly associated with nighttime activity: dogs were more active on weekends regardless of treatment. While these null results with regard to treatment are consistent with the previous study, they are in contrast to the reports of owners; using the SNoRE questionnaire, owners *were* able to detect a difference in sleep quality when their dogs were on meloxicam. In the previous study, average activity per hour over the nighttime period showed a small, but non-significant, decrease in activity with meloxicam treatment^10^. By applying an FLM approach, we were able to detect this difference in dogs’ overnight activity and found activity data matched owners’ assessments. The most significant differences were found during the overnight period from approximately 11 pm-6 am; dogs were less active (and possibly sleeping) when receiving meloxicam rather than placebo. The 11 pm–6 am time interval corresponds to the time that the majority of owners designated as “night-time;” it also fully encompasses the period from 12 am-5 am that was designated as “night-time” for all owners in the previous study^10^. A smaller, though significant, difference was seen between approximately 7 pm and 8 pm, where dogs receiving meloxicam were more active than dogs receiving placebo; for the majority of the evening, dogs were not significantly different. The lack of difference in the evening may be due to the mediating effect of owner interaction during the evening hours. Another possibility is that the owners were instructed to give the medication in the evening, but the exact timing was not recorded. It is possible that the lack of difference between placebo and NSAID in the evening was due to decreased effectiveness towards the end of the 24-hour dosing interval. In contrast, dogs receiving placebo were significantly less active during the morning hours between 7 and 11 am. This finding fits with work from other species, including humans, showing increased daytime sleepiness following poor quality sleep^17^, and an increase in the pain-alleviating effect of sleep during the first half of the day^7^.

The findings from this study support the use of the SNoRE questionnaire as an owner-completed outcome for sleep quality in dogs with chronic pain. Our results support criterion validity of the SNoRE. The items from the SNoRE questionnaire that were most able to distinguish a positive effect of meloxicam on sleep were specifically regarding low-level movements such as twitching and dreaming. Indeed, dreaming was rated as significantly different from baseline while taking meloxicam, and change on this measure was significantly different between meloxicam and placebo. Unfortunately, we do not currently know what algorithms to apply to our activity data to be able to distinguish the types of movements being captured; future work is necessary to distinguish movements associated with dreaming or twitching from other types of movement. Non-steroidal anti-inflammatory medications, like meloxicam, are considered generally sleep-neutral in humans, with no effect on REM sleep^18^. Future work is needed to understand what owners are classifying as “dreaming” and how this is associated with pain.

This study represents the first application of FLM to sleep in dogs, and the first application of FLM to activity data used to measure the effects of analgesics in chronic pain. Recognition of the impact of dogs as naturally-occurring models of disease is increasing^8,19,20^; as such, interest in dog sleep is growing. An area of growing research is the connection between chronic pain, sleep disturbances, and cognitive impairment in people^21,22^. As with people, sleep has demonstrated importance in learning and memory consolidation in dogs^23^. It is likely that sleep impairment due to chronic pain would have similarly disruptive effects on canine cognition; this is an area of future research. Other chronic diseases in dogs, such as cognitive dysfunction, are associated with changes in sleep-wake cycle^24^; characterization of the normal sleep-wake cycle in pet dogs, and further, the relationship between pet and caregiver sleep patterns, would be a valuable contribution to the field of veterinary and comparative medicine. A comparison of overnight activity between dogs with osteoarthritis and age matched controls remains a gap in the field; such a comparison would allow us to determine the extent of baseline sleep disturbances in dogs with chronic pain compared to those without. However, the current study supports the hypothesis that dogs with chronic pain have disturbances in sleep that are relieved when they receive adequate pain management. Each dog served as their own control, increasing our confidence that, rather than age or group-level differences between dogs, these differences were due to the treatment. This study also provides additional support for the applicability of dogs as a model of OA-associated induced and spontaneous pain in humans^25^, especially with an objective measure used commonly in studying human sleep^26,27^ and a protocol for analysis of the data.

In summary, this study has demonstrated improved sleep quality due to pain relief in a spontaneous canine model of osteoarthritis pain. It has also supported two important findings: first, the SNoRE questionnaire has criterion validity and appears to be useful in detecting the alleviation of sleep disturbances associated with chronic pain in dogs; second, FLM is a more sensitive analytic technique for activity data in dogs—this technique was able to detect a difference in activity patterns when traditional summary techniques were not. These results further expand the translational potential of client-owned dogs with osteoarthritis pain; they are a model of spontaneous pain-associated sleep disturbance and could be involved in the evaluation of putative analgesics.

## Methods

### SNoRE study

Experimental details for the SNoRE study have been previously described^10^. This was a double-masked, placebo-controlled crossover study design. Client-owned dogs (n = 15) that met rigorous inclusion and exclusion criteria received meloxicam and placebo in random order: meloxicam or placebo for two weeks, followed by a one-week washout, followed by two more weeks of meloxicam or placebo. Meloxicam was dosed at 0.2 mg/kg by mouth on the first day, followed by 0.1 mg/kg once daily; placebo was volume matched and visually identical. Owners were instructed to administer the meloxicam/placebo treatments in the evening (at approximately 6 pm) each day of the study. Dogs wore accelerometers (Actical, Philips Respironics) to collect data each minute on spontaneous activity. Owners kept a sleep-diary and noted the times when they went to bed in the evening and rose in the morning. This allowed activity data to be classified as night-time activity (NTA) or day-time activity (DTA) for use in the model. Owners completed a series of clinical metrology instruments at various timepoints in the study. For the purposes of the current study, data are included from the Canine Brief Pain Inventory (CBPI^28^) completed on Day 0, and the Sleep and Night Time Restlessness Evaluation (SNoRE^10^) completed on Days 0, 14, 21, 28, and 35. This 6-item questionnaire evaluates sleep quality in dogs over a 7-day period. This study was approved by the North Carolina State University Institutional Animal Care and Use Committee (approval #07-188-O); all experiments were performed in accordance with relevant guidelines and regulations. All dog owners were over 18 years old and provided written informed consent.

### Data analysis

To replicate previous findings, we first analyzed activity data using a linear mixed-effects model, with the covariates of age, weight, sex, CBPI pain sub-score, and treatment (meloxicam or placebo). Based on previous work showing a difference in weekend and weekday activity patterns in dogs^29^, we added this as a covariate for our linear model. As functional data analysis is performed across a 24-hour period, we repeated our linear model for daytime activity (not previously reported).

Functional linear modeling (FLM) is specifically designed for actigraphy time-series data analysis. We used it to statistically characterize and illustrate 24-hour sleep-wake patterns of the same dogs that were either given a placebo or drug. The strength of this approach is that it measures raw activity counts within and between samples and avoids summary statistics that can mask differences across groups^16^. Therefore, we applied a non-parametric permutation test method in the R package “actigraphy”^30^, as it does not rely on distributional assumptions. Significance was calculated by counting the proportion of permutation *F* values that are larger than the *F* statistics for the observed pairing. Here, we used the point-wise test (with 500 permutations; bspline method) which provides a curve that represents the proportion of permutation *F* values that are larger than the *F* statistic for the observed pairing at each point in the time series^16^.

Individual items and total scores on the SNoRE questionnaire were analyzed using matched pairs t-tests to compare the meloxicam and placebo treatment (Days 14 and 35) to baseline (Day 0), and to each other. To adjust for these multiple comparisons, a critical p-value of 0.016 was considered significant.
